# Co-transplantation of mesenchymal stem cells improves spermatogonial stem cell transplantation efficiency in mice

**DOI:** 10.1186/s13287-018-1065-0

**Published:** 2018-11-21

**Authors:** Prashant Kadam, Elissavet Ntemou, Yoni Baert, Sven Van Laere, Dorien Van Saen, Ellen Goossens

**Affiliations:** 1 Biology of the Testis (BITE) Laboratory, Department of Reproduction, Genetics and Regenerative Medicine, Vrije Universiteit Brussel (VUB), Laarbeeklaan 103, 1090 Brussels, Belgium; 2 Biostatistics and Medical Informatics (BISI) Research Group, Department of Public Health, Vrije Universiteit Brussel (VUB), Laarbeeklaan 103, 1090 Brussels, Belgium

**Keywords:** Fertility restoration, Infertility, Mesenchymal stem cells, Spermatogonial stem cells, Transplantation

## Abstract

**Background:**

Spermatogonial stem cell transplantation (SSCT) could become a fertility restoration tool for childhood cancer survivors. However, since in mice, the colonization efficiency of transplanted spermatogonial stem cells (SSCs) is only 12%, the efficiency of the procedure needs to be improved before clinical implementation is possible. Co-transplantation of mesenchymal stem cells (MSCs) might increase colonization efficiency of SSCs by restoring the SSC niche after gonadotoxic treatment.

**Methods:**

A mouse model for long-term infertility was developed and used to transplant SSCs (SSCT, *n* = 10), MSCs (MSCT, *n* = 10), a combination of SSCs and MSCs (MS-SSCT, *n* = 10), or a combination of SSCs and TGFß1-treated MSCs (MSi-SSCT, *n* = 10).

**Results:**

The best model for transplantation was obtained after intraperitoneal injection of busulfan (40 mg/kg body weight) at 4 weeks followed by CdCl_2_ (2 mg/kg body weight) at 8 weeks of age and transplantation at 11 weeks of age. Three months after transplantation, spermatogenesis resumed with a significantly better tubular fertility index (TFI) in all transplanted groups compared to non-transplanted controls (*P* < 0.001). TFI after MSi-SSCT (83.3 ± 19.5%) was significantly higher compared to MS-SSCT (71.5 ± 21.7%, *P* = 0.036) but did not differ statistically compared to SSCT (78.2 ± 12.5%). In contrast, TFI after MSCT (50.2 ± 22.5%) was significantly lower compared to SSCT (*P* < 0.001). Interestingly, donor-derived TFI was found to be significantly improved after MSi-SSCT (18.8 ± 8.0%) compared to SSCT (1.9 ± 1.1%; *P* < 0.001), MSCT (0.0 ± 0.0%; *P* < 0.001), and MS-SSCT (3.4 ± 1.9%; *P* < 0.001). While analyses showed that both native and TGFß1-treated MSCs maintained characteristics of MSCs, the latter showed less migratory characteristics and was not detected in other organs.

**Conclusion:**

Co-transplanting SSCs and TGFß1-treated MSCs significantly improves the recovery of endogenous SSCs and increases the homing efficiency of transplanted SSCs. This procedure could become an efficient method to treat infertility in a clinical setup, once the safety of the technique has been proven.

**Electronic supplementary material:**

The online version of this article (10.1186/s13287-018-1065-0) contains supplementary material, which is available to authorized users.

## Background

Advanced cancer treatments have resulted in more than 80% survival rate of childhood cancer patients [1, 2]. However, a childhood cancer survivor study reported that 46% of these patients would become infertile, while 18% of their siblings will experience fertility problems in adulthood [3]. As these boys lack active spermatogenesis at the time of cancer diagnosis, they cannot benefit from sperm freezing. Testicular tissue banking in combination with spermatogonial stem cell transplantation (SSCT) has been proposed as a possible fertility preservation strategy for these patients [4, 5]. However, this procedure shows a low homing efficiency of SSCs [6] and a deficient epigenetic reprogramming after SSCT [7], which could be attributed to the damaged supportive microenvironment. Substantial evidence shows that both chemo- and radiotherapy affect not only the SSCs but also the niche cells (Sertoli, Leydig, and peritubular cells) [8–10]. Transplanting only SSCs might thus be inefficient to restore fertility after gonadotoxic treatment. To improve the efficiency of the procedure, co-transplantation of supporting cells or factors may be considered.

Mesenchymal stem cells (MSCs) are multipotent adult stem cells with proven regenerative potential residing in all organs of the body [11]. Paracrine growth factors secreted by MSCs have shown anti-apoptotic, anti-inflammatory, and anti-oxidative properties [12, 13]. Co-transplantation of cardiac stem cells and MSCs has shown highly promising results in the cell therapy for myocardial infarction and chronic heart failure [14]. Also, in the treatment of diabetes, co-transplantation of islets of Langerhans with MSCs showed better remodeling, structural organization, and revascularization compared to islets transplanted alone [15]. Moreover, hyperglycemia was reverted to normal in 92% of mice co-transplanted with MSCs compared with 42% of those transplanted with islets alone. Thus, co-transplantation improves not only the structural organization but also the organ functionality [15]. MSC-based cell and gene therapies have already been successful in both pre-clinical and clinical studies for the treatment of disorders in visceral organs (heart, liver, kidney, lung, and pancreas), the musculoskeletal system, and the nervous system (for review: [16]).

The benefit of transplanting SSCs together with MSCs has been hypothesized [17] but not yet investigated. Nevertheless, in various animal models, MSCs from different sources (bone marrow, adipose tissue, and umbilical cord) have been transplanted as an alternative to SSCs. However, this was not very successful. MSCs did not transdifferentiate towards germ cells and, therefore, spermatogenesis could not be restored. However, importantly, endogenous spermatogenesis was re-established in all experiments [18–22]. This regenerative potential might be the effect of paracrine factors secreted by the transplanted MSCs. In vitro induction of bone marrow MSCs from ram with TGFβ1 for 21 days resulted in the upregulation of the germ cell-specific genes *DDX4*, *PIWIL2*, *ITGb1*, *OCT4*, and *DAZL*. These TGFβ1-treated MSCs were found to survive, home, and form colonies after transplantation into ram testes and also expressed the germ cell marker PGP9.5/UCHL1. Although MSC-derived spermatogenesis was not achieved, recovery of endogenous spermatogenesis was reported [23].

We hypothesize that co-transplanting MSCs along with SSCs might improve SSCT efficiency by restoring the testicular niche. To investigate the regenerative potential of MSCs, we optimized an infertility model representing the impaired somatic environment. Next, we co-transplanted SSCs with either native MSCs or TGFβ1-treated MSCs and compared this with the transplantation of only SSCs or MSCs.

## Methods

### Optimization of the mouse model for infertility

**Fig. 1:**
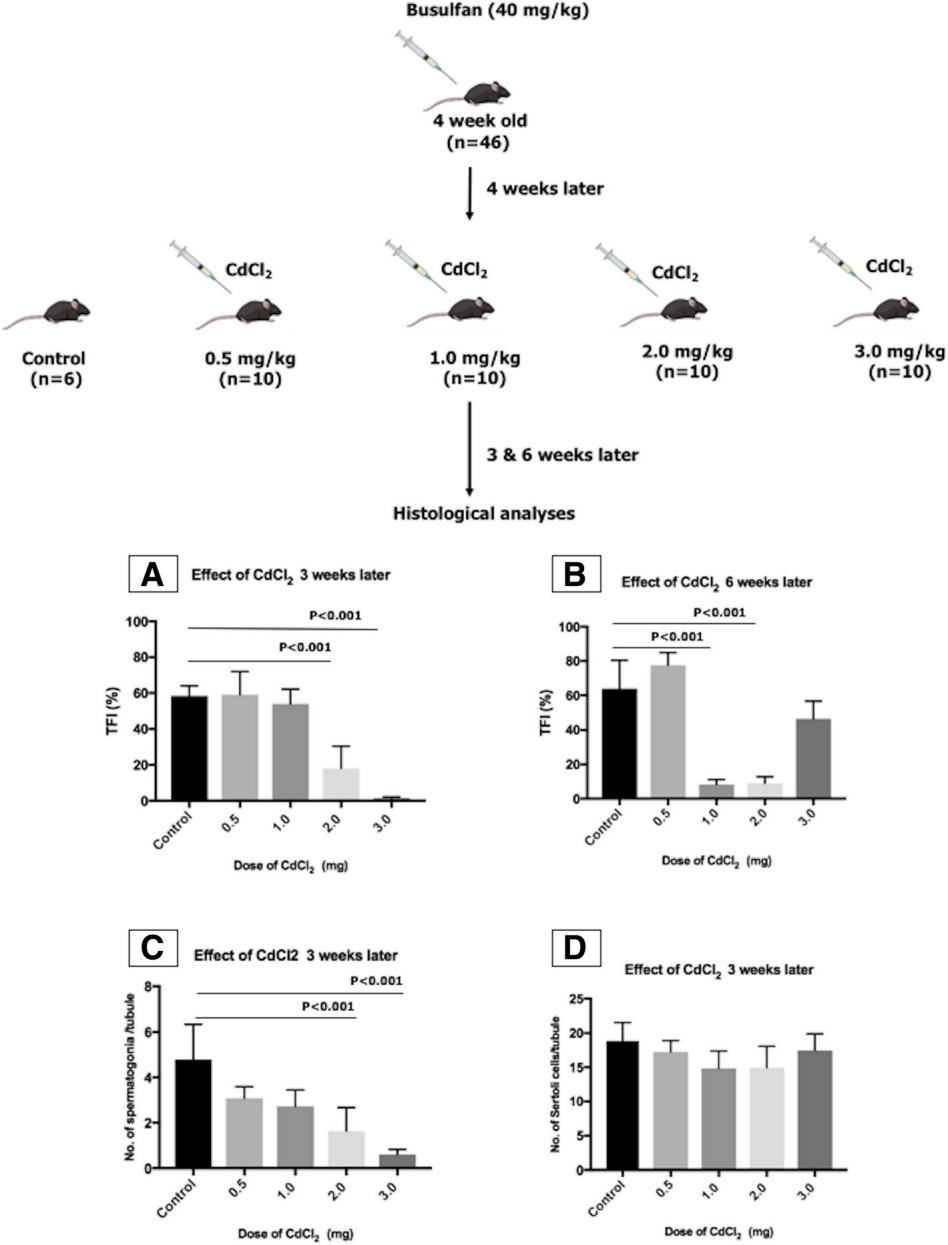
Optimization of the mouse model for infertility. Several doses of CdCl_2_(0.5, 1.0, 2.0, and 3.0 mg/kg body weight) were tested in combination with busulfan (40 mg/kg body weight). TFI was assessed at two different time points after CdCl_2_ injection: (A) at week 3 and (B) week 6. (C) Graph showing quantitative analysis of spermatogonia per seminiferous tubule and (D) Sertoli cells per seminiferous tubule after CdCl_2_ treatment 3 weeks after injection

Three-week-old C57BL/6J mice were purchased from Charles River Laboratories, Paris, France, and used as recipients. In order to deplete endogenous spermatogenesis, mice (*n* = 46) were given busulfan intraperitoneally at a dose of 40 mg/kg body weight at 4 weeks of age. To ensure the depletion of Sertoli cell function, 4 weeks later, cadmium chloride (CdCl_2_), which has been shown to induce apoptosis of Sertoli cells in vitro [24], was administered at different doses (0.5, 1.0, 2.0, and 3.0 mg/kg body; *n* = 10 in each group). Six mice treated only with busulfan were used as control (Fig. 1). Three weeks after CdCl_2_ injections, half of the mice from each group were sacrificed by cervical dislocation, while the other half was sacrificed after 6 weeks. Their testes were collected in Dulbecco’s modified Eagle’s medium/F12 (DMEM/F12; 31330-038; Invitrogen, Merelbeke, Belgium). The tunica albuginea was removed, and the testicular tissue was fixed in acidified formol alcohol fixative (10056710; Labonord, Rekkem, Belgium) for at least 1 h. The tissue was placed overnight in a vacuum infiltration processor bath (Bayer, Diegem, Belgium) and embedded in paraffin the next morning. From each testis, 5-μm-thick serial sections were cut with a microtome (SM2010R; Leica, Brussels, Belgium). Serial cross sections at three different depths (at least 100 μm shift) were blindly analyzed. Histological evaluations were done with hematoxylin and eosin (HE) to assess the overall tubular fertility index (TFI; percentage of tubules containing spermatogenesis). Seminiferous tubules were analyzed by immunofluorescence staining by assessing the number of spermatogonia (UCHL1^+^) and Sertoli (SOX9^+^) cells per round tubule (Additional file 1 and Additional file 2: Figure S1C–D). A tubule was considered to be round if the ratio between the longest diameter of the tubule and the diameter perpendicular to it was less than 1.5.

### Mesenchymal stem cell culture

**Fig. 2:**
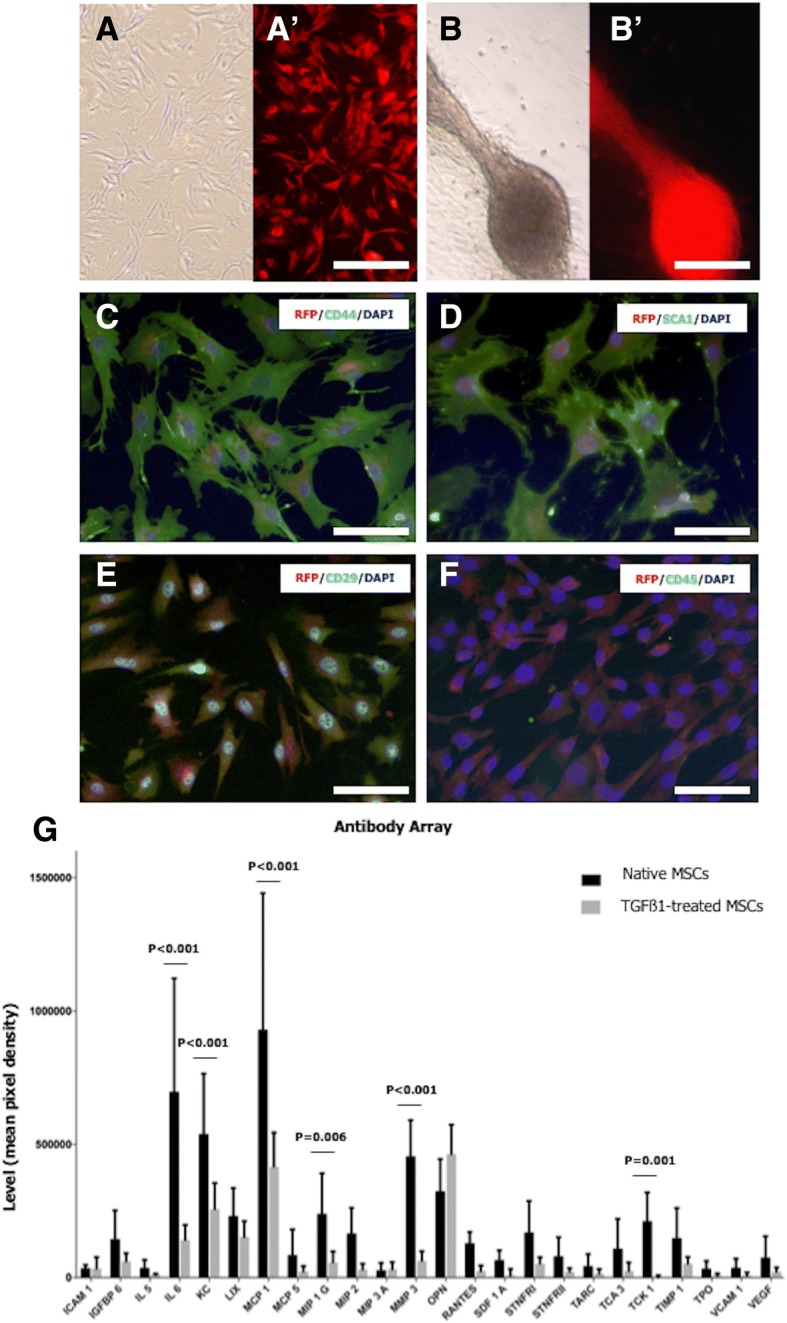
Mesenchymal stem cell culture. RFP^+^ MSCs were cultured in the absence (**A**, **A′**) or presence of TGFß1 (**B**, **B′**) for 15–21 days. MSCs showed a spindle-shaped morphology (**A** and **B** bright field in; **A′** and **B′** fluorescence microscope with Texas red filter). TGFß1-treated MSCs occasionally showed spheroid-like colonies. MSCs were positive for CD44 (**C**), SCA1 (**D**), and CD29 (**E**) and negative for CD45 (**F**). (**G**) Cytokine secretion profiles were obtained by incubating antibody array membranes with conditioned media from TGFß1-treated and non-treated MSC cultures (at 3 weeks). **A**, **A′**, **B**, **B′** Scale bar = 200 μm. **C**–**F** Scale bar = 100 μm

Red fluorescent protein (RFP)-transfected C57BL/6 mouse bone marrow MSCs (MUBMX-01201; Cyagen Biosciences, CA, USA) were cultured in T25 cell culture flasks (690175; Greiner Bio-One Vilvoorde, Belgium) at the density of 1 × 10^6^ cells/flask with and without 10 ng/ml TGFβ1 (P04202; R&D Systems, Minneapolis, USA) in OriCell™ mouse MSC basal medium (MUXMX-90011; Cyagen Biosciences, CA, USA) in a humidified incubator with 5% CO_2_ at 37 °C for 15 to 21 days (Fig. 2A, B, Additional file 3). The medium was changed every third day, and cells were passaged after reaching 80% confluency. For transplantation experiments, cells were used at passages 5 or 6.

### Antibody array

Antibody arrays targeting 96 mouse cytokines (ab193659; Abcam, Cambridge, UK) were used to identify and quantify paracrine factors secreted at the end of the culture in order to compare cytokine production in native MSCs and TGFß1-treated MSCs (Additional file 4: Figure S3 and Additional file 5: Table S1). The culture medium of four biological replicates was collected and subjected to the antibody array assay as per the manufacturer’s instructions. Chemiluminescence was detected with a ChemiDocTM MP Imaging System (Bio-rad). Densitometry was performed using Bio-Rad’s Image Lab v.5.2.1 software.

Relative levels of secretion were calculated as the average of the sum of signal intensities for each marker of interest minus the average of the sum of the signal intensities of the corresponding blank control spots. Normalization was performed by defining one array as the reference to which the other arrays were normalized from the average of the sum of the signal intensities belonging to the positive control spots. After that, for each target, the average of the sum of the signal intensities of the corresponding medium control was subtracted. We followed very rigorous inclusion criteria, and only cytokines with signal intensities above 15,000 in all four biological replicates following this correction were considered. Finally, the secretion levels observed in cultured MSCs were compared with those of TGFß1-treated MSCs.

### Testicular cell isolation and cryopreservation

Pre-pubertal GFP^+^ F1-hybrid pups (5–7 days), obtained by crossing male inbred C57BL with female inbred SV129 green fluorescent protein (GFP) in the VUB animal facility, were used as donors. Testicular cells were isolated from 20 donor testes, pooled and cryopreserved at a concentration of 1–2 × 10^6^ cells/ml using a slow freezing protocol [25]. Concentration and viability of the cell suspensions were assessed with a Tali® image-based cytometer (T10796; Life Technologies, Gent, Belgium) after fresh cell isolation and after freeze-thawing.

### Transplantation experiments

**Fig. 3:**
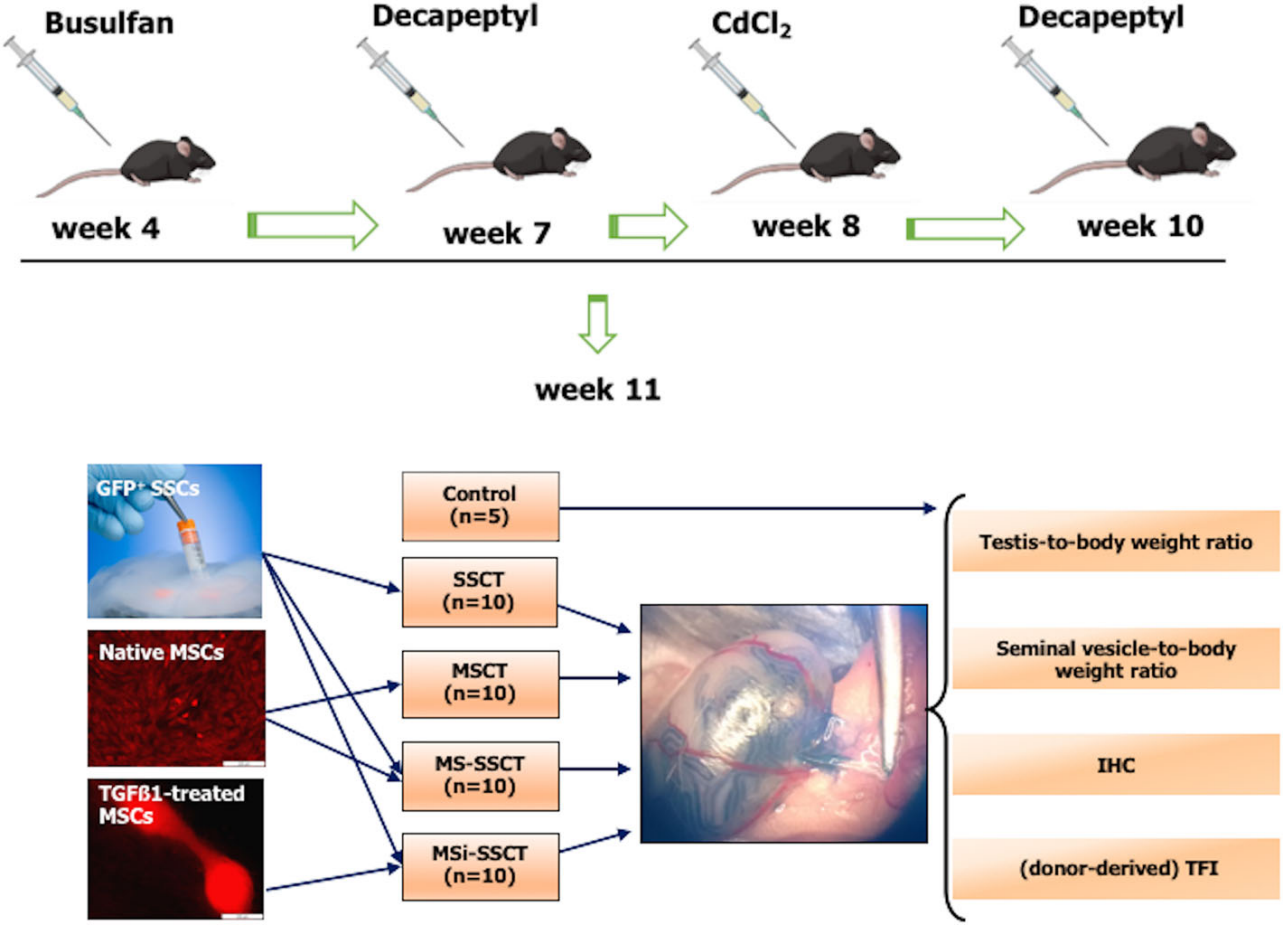
Transplantations. Transplantations were performed in GFP^−^ busulfan- and CdCl_2_-treated mice using SSCs from 5- to 7-day-old GFP^+^ mice and/or RFP^+^ MSCs. SSCT spermatogonial stem cell transplantation (*n* = 10), MSCT mesenchymal stem cell transplantation (*n* = 10), MS-SSCT transplantation of mesenchymal stem cells together with spermatogonial stem cells (*n* = 10), MSi-SSCT transplantation of TGFß1-treated mesenchymal stem cells together with spermatogonial stem cells (*n* = 10). Control (*n* = 5) mice received busulfan and CdCl_2_ but were not transplanted. Decapeptyl (4.26 mg/kg) was injected subcutaneously 1 week before CdCl_2_ injection and secondly 1 week before transplantations

Based on the results from the optimization study, recipient mice (*n* = 45) were prepared for transplantation by injecting busulfan (40 mg/kg) and CdCl_2_ (2 mg/kg) intraperitoneally. Mice were injected twice (1 week before CdCl_2_ injection and 1 week before transplantation) with a subcutaneous dose of the GnRH agonist Decapeptyl (4.26 mg/kg; 0.1 mg; Ipsen, Paris, France) to improve homing and colonization of transplanted cells [26] (Fig. 3). Transplantations were performed under a stereomicroscope as previously described [25]. Mice were anesthetized with a mixture of an intraperitoneal injection (150–200 μl) of 75 mg/kg ketamine (Ketamidor®; Ecuphar, Oostkamp, Belgium) and 1.0 mg/kg medetomidine (Medetor®; Virbac Animal Health, Burgdorf, Germany). A subcutaneous dose (50 μl, 5 mg/kg body weight) of the analgesic meloxicam (Metacam®; Boehringer Ingelheim, Vetmedica GmbH, Ingelheim am Rhein, Germany) was administrated pre-operatively and for 2 days post-operatively. The surgical area was prepared by clipping abdominal hair and disinfected with cedium chlorhexidini alcoholicus 0.5% (BE351513; Laboratoires Gifrer Barbezat, France). The abdomen was incised, and the testes were exteriorized.

After thawing and assessing concentration and viability (with a Tali® image-based cytometer (T10796; Life Technologies, Gent, Belgium), GFP^+^ donor cells were resuspended in injection medium [(DMEM/F12, 10% penicillin/streptomycin (15140-122; Life Technologies, Merelbeke, Belgium) containing 4% fetal calf serum (10500-056; FCS, Life Technologies)] to obtain a concentration of 10–20 × 10^6^ cells/ml.

The experiment consisted of four different transplantation groups and one control group. The first transplantation group (*n* = 10) received SSCs (SSCT). The second group (*n* = 10) received MSCs (MSCT). The third group (*n* = 10) received both SSCs and MSCs (MS-SSCT), and the fourth group (*n* = 10) received SSCs and TGFß1-treated MSCs (MSi-SSCT). Ten microliters or 2 × 10^5^ cells were injected per testis. For groups 3 and 4, the two cell types were mixed in 1:1 volume (SSCs/MSCs or SSCs/TGFß1-treated MSCs), keeping the total cell concentration constant. Immediately after transplantation, mice received a subcutaneous dose of 5 mg/kg (50 μl) of enrofloxacin (Baytril®; Bayer, Diegem, Belgium). The control group (*n* = 5) was treated with busulfan, CdCl_2_, and Decapeptyl but did not undergo transplantation (Fig. 3).

### Immunohistochemistry and histological analysis

Three months after transplantation, 30 serial cross sections per testis (with a 100 μm shift between each slide) were blindly analyzed to assess the overall TFI (percent of tubules containing spermatogenesis) and the donor-derived TFI (percent of tubules containing donor-derived spermatogenesis) [27]. Double immunofluorescent staining was performed for RFP (MSCs) and MVH (Germ cell), SOX9 (Sertoli cell), or STAR (Leydig cell) to evaluate the expression of testicular cell markers by transplanted MSCs (Additional file 1 and Additional file 6).

### Statistical analyses

Statistical analysis was done using the software package SPSS (IBM, SPSS Statistics Version 25, IBM Corporation, Somers, NY, USA). All data are presented as means ± standard deviation (SD). A one-way analysis of variance (ANOVA) was used in order to find differences between different doses or treatments. A post hoc test with Bonferroni correction was used in order to find pairwise significant differences. Normality of the data was verified using the Kolmogorov-Smirnov test. When normality of the data did not hold, the Kruskal-Wallis test was used as an alternative to the ANOVA test. A difference between the level of cytokines (mean pixel densities) was analyzed using the Holm-Šídák multiple comparison tests for comparing the treated with the non-treated MSCs. *P* values < 0.05 are considered statistically significant. Graphs were prepared using GraphPad Prism 5 (GraphPad Software, Inc., La Jolla, CA).

## Results

### Optimization of the mouse model for infertility

Because endogenous spermatogenesis recovers rather quickly in the generally used transplantation model, we aimed to create a mouse model which better represented the clinical condition (no recovery of endogenous spermatogenesis) by damaging the SSC niche. More than 80% of the tubules were without spermatogenesis after 3 weeks in the groups that received 2 or 3 mg/kg CdCl_2_ (Fig. 1A). However, after 6 weeks, the regeneration of spermatogenesis was observed for the 3 mg CdCl_2_ group (Fig. 1B). Moreover, severe degeneration and atrophy of seminiferous tubules were evident in both 2 and 3 mg CdCl_2_ groups (Additional file 2: Figure S1A-B). Immunofluorescent staining for UCHL1 showed a decrease in the number of spermatogonia per seminiferous tubule with increasing dose of CdCl_2_ (Fig. 1 and Additional file 2: Figure S1C). However, immunofluorescent staining for SOX9 showed that the number of Sertoli cells per seminiferous tubule was unaffected (Fig. 1D and Additional file 2: Figure S1D). Both 2 and 3 mg/kg CdCl_2_ cleared 80% of the tubules from germ cells. But since two mice died in the 3 mg/kg CdCl_2_ group, the lower dose of 2 mg/kg CdCl_2_ was used for the transplantation experiments.

### Culture and characterization of MSCs

When cultured in vitro, MSCs showed a long spindle-shaped and fibroblast-like morphology (Fig. 2A and A′). However, occasionally, TGFβ1-treated MSCs also showed spheroid-like colonies at 15–21 days of culture (Fig. 2B and B′). After 3 weeks in culture, cells still showed RFP expression and expressed the MSC markers CD44, SCA1, and CD29, but were negative for CD45 (Fig. 2C–F, Additional file 3: Figure S2 E–H). The expression pattern of these markers remained the same throughout the culture period in both the control and TGFβ1-treated condition.

The antibody array showed that 24 out of 96 cytokines were detected in both the TGFß1-treated and non-treated group. TGFß1 treatment resulted in significantly lower expression of IL6 (*P* < 0.001), MCP1 (*P* < 0.001), MMP3 (*P* < 0.001), TCK1 (*P* = 0.001), KC (*P* < 0.001), and MIP1G (*P* = 0.006) compared to non-treated MSCs (Fig. 2G, Additional file 4: Figure S3, Additional file 5: Table S1).

### Transplantations

**Fig. 4:**
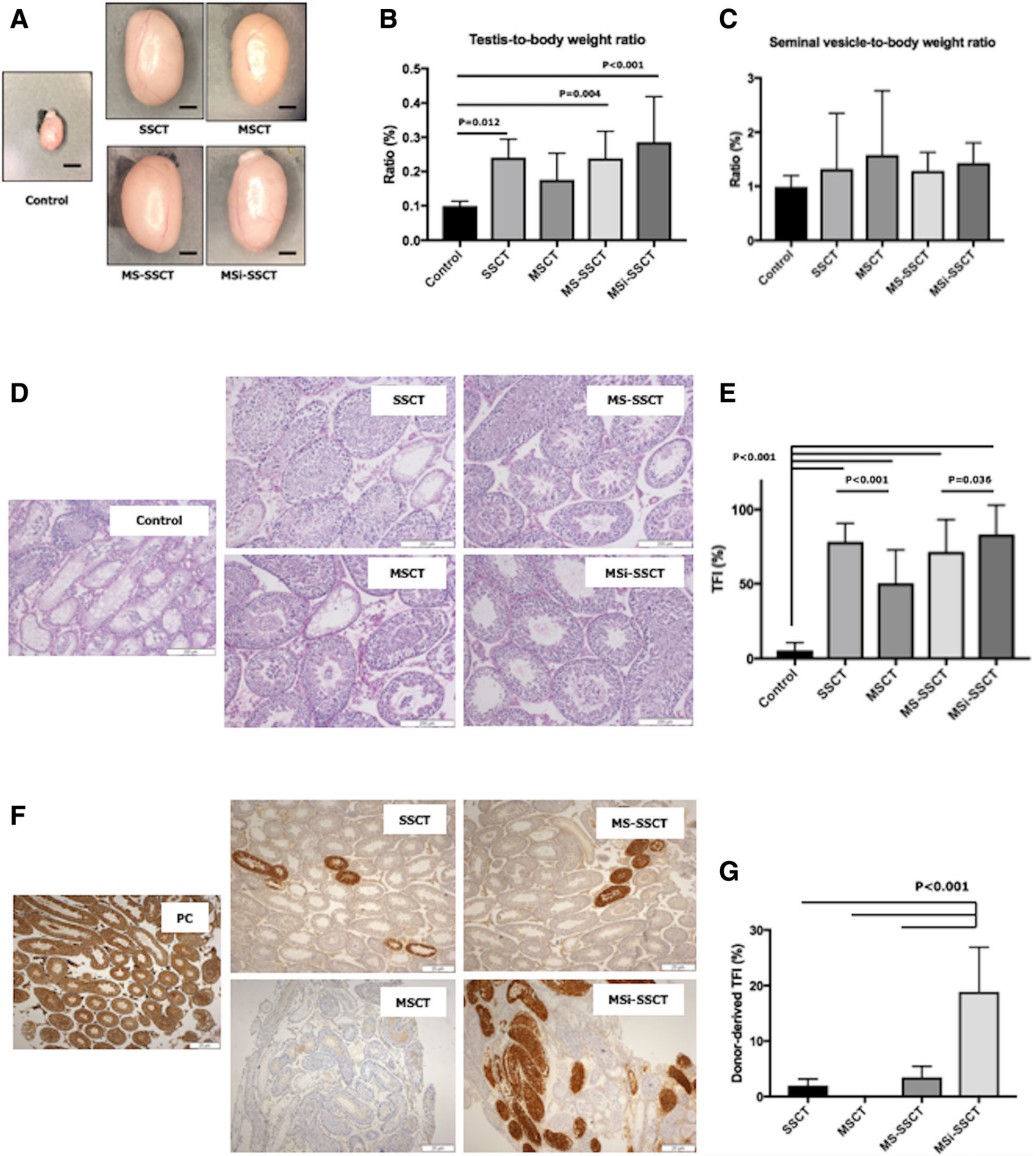
Testicular histology 3 months after transplantation. The significant increase of testis size (**a**) and testis-to-body weight ratio (**b**) was found in transplanted groups compared to controls (control vs. SSCT, *P* = 0.012; control vs. MS-SSCT, *P* = 0.004, and control vs. MSi-SSCT, *P* < 0.001). However, no difference was observed for the seminal vesicle-to-body weight ratio (**c**). Resumption of spermatogenesis was found to be significantly improved in all transplanted groups compared to controls (*P* < 0.001) (**d**, **e**); Tubular fertility index (TFI) did not differ between SSCT and MSi-SSCT groups (**e**). Donor-derived spermatogenesis was confirmed with immunohistochemistry for GFP (**f**). Donor-derived TFI was found to be significantly different between SSCT and MSi-SSCT (*P* < 0.001), MSCT and MSi-SSCT (*P* < 0.001), and MS-SSCT and MSi-SSCT (*P* < 0.001) (**g**). SSCT, spermatogonial stem cell transplantation; MSCT, mesenchymal stem cell transplantation; MS-SSCT, transplantation of mesenchymal stem cells together with spermatogonial stem cells; MSi-SSCT, transplantation of TGFß1-treated mesenchymal stem cells together with spermatogonial stem cells. **a** Scale bar = 2 mm

Three months after transplantation (Fig. 3), mice were sacrificed and the testes were collected. In the MSCT group, three mice died shortly after the transplantation. A significant increase of testis size and testis-to-body weight ratio was found in transplanted groups compared to controls (Fig. 4a, b) (control vs. SSCT: *P* = 0.012; control vs. MS-SSCT: *P* = 0.004; control vs. MSi-SSCT: *P* < 0.001). However, no difference was observed for the seminal vesicle-to-body weight ratio (Fig. 4c). Resumption of spermatogenesis was found to be significantly improved in all transplanted groups compared to controls (*P* < 0.001). TFI did not differ significantly between SSCT and MS-SSCT, nor with MSi-SSCT. However, interestingly, a significantly lower TFI was seen in MSCT compared to SSCT (*P* < 0.001) (Fig. 4d, e). MSi-SSCT was significantly better when compared to MS-SSCT (*P* = 0.036). Overall, SSCT and MSi-SSCT gave the best results. Donor-derived spermatogenesis was confirmed with immunohistochemistry for GFP (Fig. 4f). Donor-derived spermatogenesis was found in 21.4% of the successfully injected testes after SSCT, in 0% after MSCT, in 12.5% after MS-SSCT, and in 50.0% after MSi-SSCT. The donor-derived TFI was found to be significantly higher in the MSi-SSCT group (18.8 ± 8.0%) compared to the SSCT (1.9 ± 1.1%; *P* < 0.001), MSCT (0.0 ± 0.0%; *P* < 0.001), and MS-SSCT (3.4 ± 1.9%; *P* < 0.001) group (Fig. 4g).

### The fate of MSCs after transplantation

**Fig. 5:**
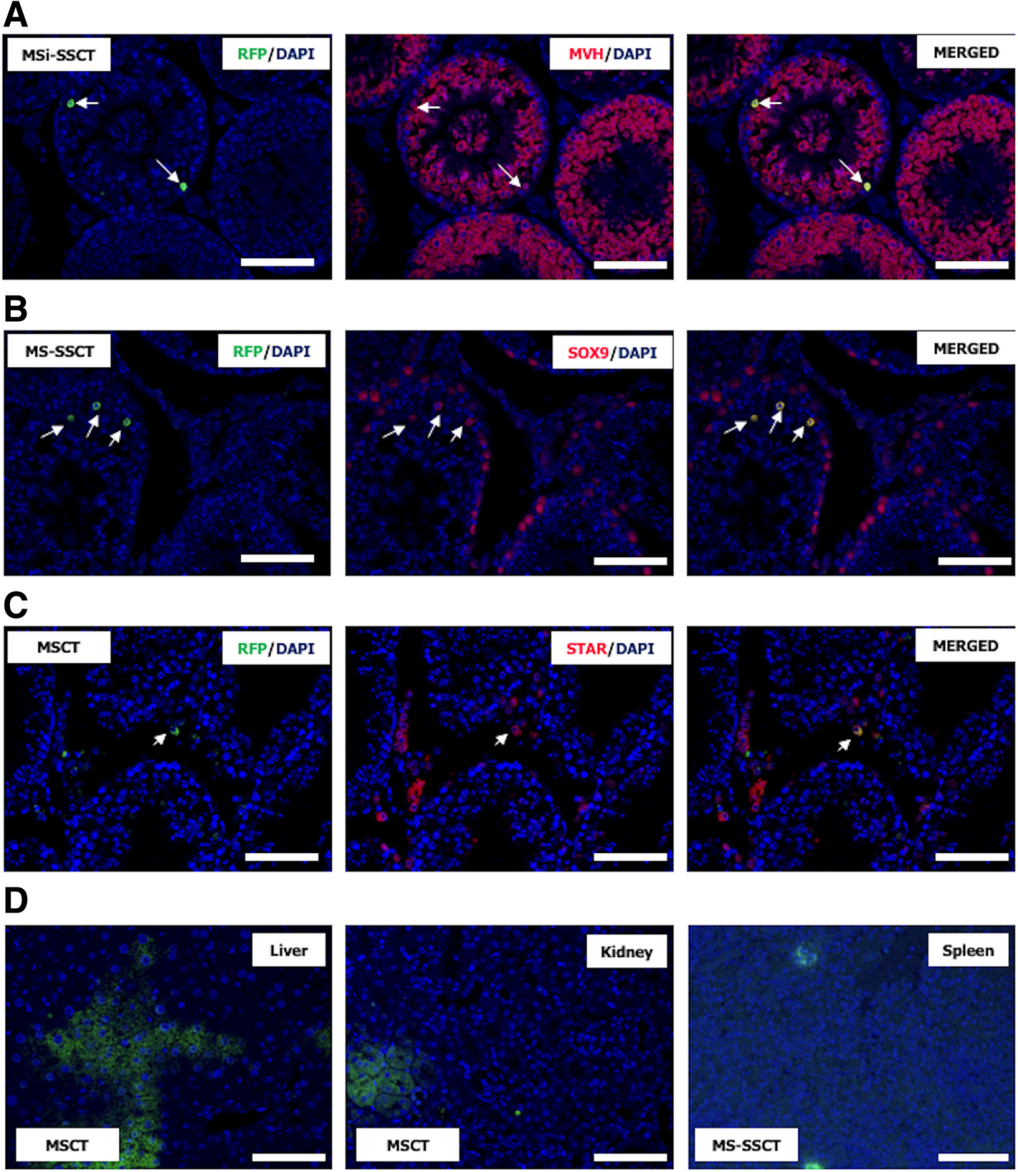
Detection of transplanted mesenchymal stem cells. **a** Transplanted RFP^+^ MSCs (arrows) expressed **a** the germ cell marker MVH in all transplantation groups (MSCT, MS-SSCT, and MSi-SSCT). **b** The Sertoli cell marker SOX9 or **c** the Leydig cell marker STAR was expressed in transplanted MSCs after MSCT and MS-SSCT, but not after MSi-SSCT. **d** RFP^+^ MSCs (green) could also be detected in the liver, kidney, and spleen. Scale bar = 50 μm

Transplanted MSCs expressed the germ cell marker MVH (Fig. 5a). The percentage of tubules with MVH^+^ MSCs after MS-SSCT (51.0 ± 24.6%) and MSi-SSCT (51.3 ± 23.2%) was found to be significantly higher compared to MSCT (24.6 ± 9.6%; *P* < 0.001). Very rarely, some tubules showed cells expressing the Sertoli cell marker SOX9 after MSCT and MS-SSCT (Fig. 5b). The Leydig cell marker STAR was found to be co-expressed with RFP (Fig. 5c) in the case of failed transplantations (interstitial injection). In the MSi-SSCT group, MSCs showed MVH expression, but none expressed SOX9 or STAR. MSCs were also found to have migrated to the visceral organs (liver, kidney, and spleen) after MSCT and MS-SSCT (Fig. 5d) but not after MSi-SSCT.

## Discussion

In human, chemo- and/or radiotherapy not only deplete SSCs but might also affect the niche cells [28]. Busulfan has been the drug of choice to prepare recipient mice for SSCT. Busulfan treatment destroys spermatogenesis by damaging the germ cells and Sertoli cells [29, 30]. However, doses lower than 40 mg/kg are found to be inconsistent as this did not result in prolonged depletion of germ cells [31] but usually result in a resumption of endogenous spermatogenesis. Alternatively, low doses of bi-lateral intra-testicular busulfan injections resulted in the better outcome and minimization of the risk of systemic toxicity [32]. Busulfan treatment followed by intratubular injection of cadmium sulfate completely destroyed the Sertoli cells but manifested inflammatory reaction increasing cellular debris [33]. Since CdCl_2_ affects not only the SSCs but also the Sertoli and Leydig cells [9, 24], we chose to use both busulfan and CdCl_2_ but through intraperitoneal route to deplete SSCs and their niche cells in order to better mimic the clinical situation. Although CdCl_2_ did not have an effect on the number of Sertoli cells, their function might have been impaired, as spermatogenic recovery was rarely seen (5% TFI; Fig. 4e).

Our results showed that transplanted MSCs started to co-express the germ cell marker MVH or the Sertoli cell marker SOX9. However, MSC-derived complete spermatogenesis was never observed. These results are in line with those obtained by Ghasemzadeh-Hasankolaei et al. in 2016. In ram, TGFß1-treated bone marrow MSCs expressed germline-specific genes and were able to survive, home, and form colonies in the testes after transplantation. Although differentiation towards mature spermatozoa could not be proven, these results confirm its critical regulatory role during spermatogenesis [23]. A similar observation was reported recently when treating acute muscle injury in mice with autologous transplantation of adipose tissue-derived MSCs [34]. Muscle regeneration was improved, but the direct differentiation of MSCs was never observed. The regenerative potential was attributed to paracrine factors secreted by MSCs [34].

Donor-derived spermatogenesis was found in 21.4% of successfully injected testes after SSCT which was significantly improved after MSi-SSCT (50%) but is low compared to our previous study (75%) [35]. The latter could be explained by the aggressive recipient preparation (busulfan and CdCl_2_) leading to a more severely damaged SSC niche. But, interestingly, we could achieve up to 19% of donor-derived TFI after MSi-SSCT, while this was only 2% in the SSCT group (9% in our previous study [35]). Moreover, in all transplanted groups, endogenous spermatogenesis was restored which proves the regenerative potential of MSCs and their supportive role in re-establishing the SSC niche. After transplantation, the testicular size and testis-to-body weight ratio were improved, showing the recovery from the toxic treatment. The seminal vesicle-to-body weight ratio did not change, which indicates a normal testosterone level [36].

Spermatogenic recovery did not differ between SSCT and MSi-SSCT. However, when only donor-derived spermatogenesis is taken into account, co-transplanting SSCs with MSCs almost doubled the SSCT efficiency (TFI 1.94% vs. 3.44%). Although complete trans-differentiation of MSCs towards spermatozoa was never observed, 50% of the tubules showed MVH^+^ MSCs after MS-SSCT and MSi-SSCT. Thus, the higher donor-derived TFI could be attributed to better homing and proliferation of SSCs due to the paracrine factors secreted by MSCs. Indeed, MSCs secrete factors that play a role in cell survival, immunomodulation, cell migration, angiogenesis, proliferation, and antioxidation [12, 13]. Stimulating effects on the niche cells and remaining endogenous SSCs could explain why transplanting MSCs alone also resulted in a better spermatogenic recovery compared to non-transplanted controls. In female mice, co-transplanting ovarian tissue and MSCs could restore fertility after chemotherapy. The use of adipose tissue-derived MSCs showed a better vascularization in the xenografted human ovarian tissue resulting in improved follicular survival, growth, and tissue oxygenation while reducing apoptosis [37].

Most importantly, the highest TFI (19%) was observed after co-transplanting SSCs with TGFß1-treated MSCs. TGFß1 treatment might have stimulated the MSCs to preferentially home to the testis (and less to other organs) to enhance SSC homing and proliferation and contribute to the regeneration of the damaged niche.

As busulfan and CdCl_2_ may affect the function of other organs as well [9, 38] and MSCs migrate to sites of injury through the lymphatic circulation [39], transplanted MSCs could be detected in the liver, kidney, and spleen after MSCT and MS-SSCT. The migratory property of MSCs makes them an ideal candidate for regenerative therapies, but the challenge is to deliver them at the expected site. SSCs do not show this migratory property. Interestingly, also in the MSi-SSCT group, we did not find transplanted MSCs in organs other than the testes. Antibody array data gave some insight on the molecular mechanisms of TGFß1 treatment. TGFß1 treatment resulted in significantly lower expression of IL6, MCP1, MMP3, TCK1, KC, and MIP1G which have been previously reported to play a role in inflammation and migration [40–43]. Inhibition of these paracrine factors by TGFß1 might have resulted in MSCs that lost their migratory property and remained in the testis which might have further contributed to restoring the SSC niche, resulting in a higher donor-derived TFI.

Tracking the cells in real-time by using time-lapse systems on living animals would provide more insight into SSC homing and MSC migration. The processes of MSC migration have been studied before by using advanced imaging techniques like bioluminescence [44] and PET-CT scan [45].

## Conclusion

Co-transplantation of SSCs together with TGFß1-treated MSCs improves fertility restoration efficiency in mice, probably because TGFß1 treatment preferentially directs MSCs to the testis where their secretion factors restore the testicular niche. Although more pre-clinical studies are necessary to gain more insight into the mechanism, MSi-SSCT has the potential to become a clinical application in the field of fertility preservation, once the reproductive safety has been proven.

## Additional files

Additional file 1: Methods (Immunocytochemistry and Immunohistochemistry). (DOCX 35 kb)

Additional file 2: **Figure S1.** The effect of CdCl_2_ on the testicular histology (A) three and (B) six weeks after CdCl_2_ injection. Sections were stained with hematoxylin and eosin. (C). Remaining spermatogonia (UCHL1^+^) could be found in each experimental group, as well as (D) Sertoli cells (SOX9^+^). (TIF 51268 kb)

Additional file 3: **Figure S2.** Prepubertal mouse testis tissue was used as a positive control for (A) UCHL1 and (B) SOX9 staining. Anti-RFP antibody was tested on (C) RFP^+^ cells (positive control) and (D) mouse testicular cells (negative control). Corresponding negative controls for (E) CD44, (F) SCA1, (G) CD29 and (H) CD45. Scale bars in A-B = 100 μm; in C-D = 50 μm; in E-H = 100 μm. (TIF 66904 kb)

Additional file 4: **Figure S3.** Antibody array: representative detection of 96 different mouse cytokines. The profiles of cytokine secretion in the conditioned media of (A) non-treated MSCs and (B) TGFß1-treated MSCs after three-week culture. (TIF 96864 kb)

Additional file 5: **Table S1.** Cytokines studied by the antibody array. (DOCX 37 kb)

Additional file 6: **Table S2.** Antibodies used for immunocytochemistry and immunohistochemistry. (DOCX 32 kb)

## Abbreviations

BSA: Bovine serum albumin
CdCl_2_: Cadmium chloride
DAPI: 4′,6-Diamidino-2-phenylindole
*DAZL*: Deleted in azoospermia like
*DDX4*: DEAD-box helicase 4
DMEM/F12: Dulbecco’s modified Eagle’s medium/F12
FCS: Fetal calf serum
GFP: Green fluorescent protein
HE: Hematoxylin and eosin
*ITGb1*: Integrin subunit beta 1
MSCs: Mesenchymal stem cells
MSCT: Mesenchymal stem cell transplantation
MSi-SSCT: A combination of SSCs and TGFß1-treated MSCs
MS-SSCT: A combination of SSC and MSC transplantation
NCS: Normal chicken serum
*OCT4*: Octamer-binding protein 4
PBS: Phosphate-buffered saline
*PIWIL2*: Piwi-like RNA-mediated gene silencing 2
RFP: Red fluorescent protein
SD: Standard deviation
SSCs: Spermatogonial stem cells
SSCT: Spermatogonial stem cell transplantation
TBS: Tris-buffered saline
TFI: Tubular fertility index
TGFß1: Transforming growth factor ß1

## References

[1] Ward E, Desantis C, Robbins A, Kohler B, Jemal A (2014). Childhood and adolescent cancer statistics, 2014. CA Cancer J Clin.

[2] Miller KD, Siegel RL, Lin CC, Mariotto AB, Kramer JL, Rowland JH (2016). Cancer treatment and survivorship statistics, 2016. CA Cancer J Clin.

[3] Wasilewski-Masker K, Seidel KD, Leisenring W, Mertens AC, Shnorhavorian M, Ritenour CW (2014). Male infertility in long-term survivors of pediatric cancer: a report from the childhood cancer survivor study. J Cancer Surviv.

[4] Picton HM, Wyns C, Anderson RA, Goossens E, Jahnukainen K, Kliesch S (2015). A European perspective on testicular tissue cryopreservation for fertility preservation in prepubertal and adolescent boys. Hum Reprod.

[5] Goossens E, Tournaye H (2013). Adult stem cells in the human testis. Semin Reprod Med.

[6] Nagano MC (2003). Homing efficiency and proliferation kinetics of male germ line stem cells following transplantation in mice. Biol Reprod.

[7] Goossens E, Bilgec T, Van Saen D, Tournaye H (2011). Mouse germ cells go through typical epigenetic modifications after intratesticular tissue grafting. Hum Reprod.

[8] Anderson RA, Mitchell RT, Kelsey TW, Spears N, Telfer EE, Wallace WHB (2015). Cancer treatment and gonadal function: experimental and established strategies for fertility preservation in children and young adults. Lancet Diabetes Endocrinol.

[9] Marettová E, Maretta M, Legáth J (2015). Toxic effects of cadmium on testis of birds and mammals: a review. Anim Reprod Sci.

[10] Tröndle I, Westernströer B, Wistuba J, Terwort N, Schlatt S, Neuhaus N (2017). Irradiation affects germ and somatic cells in prepubertal monkey testis xenografts. Mol Hum Reprod.

[11] Ullah I, Baregundi Subbarao R, Rho G-J (2015). Human mesenchymal stem cells - current trends and future prospective. Biosci Rep.

[12] Maumus M, Jorgensen C, Noël D (2013). Mesenchymal stem cells in regenerative medicine applied to rheumatic diseases: role of secretome and exosomes. Biochimie.

[13] Liang X, Ding Y, Zhang Y, Tse H-F, Lian Q (2014). Paracrine mechanisms of mesenchymal stem cell-based therapy: current status and perspectives. Cell Transplant.

[14] Cambria E, Pasqualini FS, Wolint P, Günter J, Steiger J, Bopp A (2017). Translational cardiac stem cell therapy: advancing from first-generation to next-generation cell types. npj Regen Med.

[15] Rackham CL, Chagastelles PC, Nardi NB, Hauge-Evans AC, Jones PM, King AJF (2011). Co-transplantation of mesenchymal stem cells maintains islet organisation and morphology in mice. Diabetologia.

[16] D’souza N, Rossignoli F, Golinelli G, Grisendi G, Spano C, Candini O (2015). Mesenchymal stem/stromal cells as a delivery platform in cell and gene therapies. BMC Med.

[17] Kadam P, Van Saen D, Goossens E (2017). Can mesenchymal stem cells improve spermatogonial stem cell transplantation efficiency?. Andrology.

[18] Nayernia K, Lee JH, Drusenheimer N, Nolte J, Wulf G, Dressel R (2006). Derivation of male germ cells from bone marrow stem cells. Lab Investig.

[19] Monsefi M, Fereydouni B, Rohani L, Talaei T (2013). Mesenchymal stem cells repair germinal cells of seminiferous tubules of sterile rats. Iran J Reprod Med.

[20] Cakici C, Buyrukcu B, Duruksu G, Haliloglu AH, Aksoy A, Isık A (2013). Recovery of fertility in azoospermia rats after injection of adipose-tissue-derived mesenchymal stem cells: the sperm generation. Biomed Res Int.

[21] Zhang D, Liu X, Peng J, He D, Lin T, Zhu J (2014). Potential spermatogenesis recovery with bone marrow mesenchymal stem cells in an azoospermic rat model. Int J Mol Sci.

[22] Tamadon A, Zare S, Rahmanifar F, Tanideh N, Ramzi M, Kuhi-Hoseinabadi O (2015). Adipose tissue-derived mesenchymal stem cells repair germinal cells of seminiferous tubules of busulfan-induced azoospermic rats. J Hum Reprod Sci.

[23] Ghasemzadeh-Hasankolaei M, Eslaminejad MB, Sedighi-Gilani M (2016). Derivation of male germ cells from ram bone marrow mesenchymal stem cells by three different methods and evaluation of their fate after transplantation into the testis. In Vitro Cell Dev Biol Anim.

[24] Chung NPY, Cheng CY (2001). Is cadmium chloride-induced inter-Sertoli tight junction permeability barrier disruption a suitable in vitro model to study the events of junction disassembly during spermatogenesis in the rat testis?. Endocrinology.

[25] Frederickx V, Michiels A, Goossens E, De Block G, Van Steirteghem AC, Tournaye H (2004). Recovery, survival and functional evaluation by transplantation of frozen-thawed mouse germ cells. Hum Reprod.

[26] Dobrinski I, Ogawa T, Avarbock MR, Brinster RL (2001). Effect of the GnRH-agonist leuprolide on colonization of recipient testes by donor spermatogonial stem cells after transplantation in mice. Tissue Cell.

[27] Paniagua R, Nistal M (1984). Morphological and histometric study of human spermatogonia from birth to the onset of puberty. J Anat.

[28] Stukenborg Jan-Bernd, Jahnukainen Kirsi, Hutka Marsida, Mitchell Rod T (2018). Cancer treatment in childhood and testicular function: the importance of the somatic environment. Endocrine Connections.

[29] Anand S, Bhartiya D, Sriraman K, Mallick A (2016). Underlying mechanisms that restore spermatogenesis on transplanting healthy niche cells in busulphan treated mouse testis. Stem Cell Rev Reports.

[30] Bhartiya D, Anand S (2017). Effects of oncotherapy on testicular stem cells and niche. Mol Hum Reprod.

[31] Kanatsu-Shinohara M, Toyokuni S, Morimoto T, Matsui S, Honjo T, Shinohara T (2003). Functional assessment of self-renewal activity of male germline stem cells following cytotoxic damage and serial transplantation. Biol Reprod.

[32] Ganguli N, Wadhwa N, Usmani A, Kunj N, Ganguli N, Sarkar RK (2016). An efficient method for generating a germ cell depleted animal model for studies related to spermatogonial stem cell transplantation. Stem Cell Res Ther.

[33] Shinohara T, Orwig KE, Avarbock MR, Brinster RL (2003). Restoration of spermatogenesis in infertile mice by Sertoli cell transplantation. Biol Reprod.

[34] Gorecka A, Salemi S, Haralampieva D, Moalli F, Stroka D, Candinas D (2018). Autologous transplantation of adipose-derived stem cells improves functional recovery of skeletal muscle without direct participation in new myofiber formation. Stem Cell Res Ther.

[35] Onofre Meza J, Faes K, Kadam P, Vicini E, Van Pelt A, Goossens E (2018). What is the best protocol to cryopreserve immature mouse testicular cell suspensions?. Reprod BioMed Online.

[36] Bartke A (1974). Increased sensitivity of seminal vesicles to testosterone in a mouse strain with low plasma testosterone levels. J Endocrinol.

[37] Manavella D D, Cacciottola L, Pommé S, Desmet C M, Jordan B F, Donnez J, Amorim C A, Dolmans M M (2018). Two-step transplantation with adipose tissue-derived stem cells increases follicle survival by enhancing vascularization in xenografted frozen–thawed human ovarian tissue. Human Reproduction.

[38] Chen X, Liang M, Wang D (2018). Progress on the study of the mechanism of busulfan cytotoxicity. Cytotechnology.

[39] Gil-Ortega M, Garidou L, Barreau C, Maumus M, Breasson Genevie L, Tavernier VE (2013). Native adipose stromal cells egress from adipose tissue in vivo: evidence during lymph node activation. Stem Cells.

[40] Golle L, Gerth HU, Beul K, Heitplatz B, Barth P, Fobker M (2017). Bone marrow-derived cells and their conditioned medium induce microvascular repair in uremic rats by stimulation of endogenous repair mechanisms. Sci Rep.

[41] Chen M-S, Lin C-Y, Chiu Y-H, Chen C-P, Tsai P-J, Wang H-S (2018). IL-1 β-induced matrix metalloprotease-1 promotes mesenchymal stem cell migration via PAR1 and G-protein-coupled signaling pathway. Stem Cells Int.

[42] Bdeir K, Gollomp K, Stasiak M, Mei J, Papiewska-Pajak I, Zhao G (2017). Platelet-specific chemokines contribute to the pathogenesis of acute lung injury. Am J Respir Cell Mol Biol.

[43] Swamydas M, Ricci K, Rego SL, Dréau D (2013). Mesenchymal stem cell-derived CCL-9 and CCL-5 promote mammary tumor cell invasion and the activation of matrix metalloproteinases. Cell Adhes Migr.

[44] Strohschein K, Radojewski P, Winkler T, Duda GN, Perka C, von Roth P (2015). In vivo bioluminescence imaging - a suitable method to track mesenchymal stromal cells in a skeletal muscle trauma. Open Orthop J.

[45] Pei Z, Zeng J, Song Y, Gao Y, Wu R, Chen Y (2017). In vivo imaging to monitor differentiation and therapeutic effects of transplanted mesenchymal stem cells in myocardial infarction. Sci Rep.

