# Supplementary material for: Co-transplantation of mesenchymal stem cells improves spermatogonial stem cell transplantation efficiency in mice
Source: Stem Cell Res Ther. 2018 Nov 21;9:317. doi: 10.1186/s13287-018-1065-0 (PMC6249754; doi:10.1186/s13287-018-1065-0)
Supplement: Supplementary file 1 — Methods (Immunocytochemistry and Immunohistochemistry). (DOCX 35 kb) [file 13287_2018_1065_MOESM1_ESM.docx]

**Additional file 1: Methods**

Immunohistochemistry for UCHL1 and SOX9

Sections were deparaffinized with xylene and dehydrated in a series of ethanol (100%, 100%, 90% and 70%). After washing in phosphate buffered saline (PBS; 70011036; Life Technologies), antigen retrieval was performed at 500 W for 4 min (in microwave) using citric acid (pH 6). After another washing step in PBS, the sections were blocked for non-specific binding with 10% normal donkey serum (NDS; JAC-017-000-121; Jackson Immunoresearch Ltd, Suffolk, England) during 30 min at room temperature (RT). The primary antibody anti-UCHL1 (Additional file 6: Table S2) was added and the sections were incubated in a humidified chamber for two hours at 4°C. A similar protocol was followed for SOX 9, except that antigen retrieval was performed at 750 W for 2 x 5 min in citric acid and sections were washed in 1 x PBS + 0.05% Tween-20 for 5 min. Moreover, additional permeabilization was done with 0.2% Triton x-100 in PBS 1h at RT. The slides were washed 5 min in 1 x PBS + 0.05% Tween-20 and blocked for non-specific binding in 3% NDS diluted in 1 x PBS for 1h at RT. Next, the slides were incubated in a humidified chamber overnight at 4° C with rabbit anti-SOX9 (Additional file 6: Table S2). After three wash steps in PBS, the next day, both UCHL1 and SOX9 slides were incubated with donkey anti-rabbit Alexa-Fluor 488 for 1h at RT. After washing the slides thrice in PBS, they were mounted with DAPI and imaged on an Olympus IX 81 inverted microscope [(Olympus, Aartselaar, Belgium) with cell^F software, version 2.8 (Olympus)] using 405 nm (DAPI) and 488 nm (UCHL1 / SOX9) filters (Additional file 2: Figure S1 C and D). Testis tissue from the pre-pubertal mouse (5-6 day old) was used as a positive control and primary antibody was omitted for negative control (Additional file 3: Figure S2 A and B).

Immunocytochemistry for MSC-specific markers

Immunocytochemistry was applied in order to characterize RFP^+^ MSCs throughout the culture. Cells were fixed with 4% paraformaldehyde for one hour at room temperature once they reached 70-80% confluency. Cells were permeabilized by 1% Triton-X100 (T8787; Sigma-Aldrich Chemie GmbH, Schnelldorf, Germany) for 30 min. Blocking of nonspecific binding sites was carried out with 10% normal goat serum (039B304; tebu-bio, Boechout, Belgium) for one hour. Overnight incubation with primary antibodies (CD44, SCA1, CD29 or CD45) (Additional file 6: Table S2) was performed at 4^º^ C. The next day, cells were washed in PBS (70011051; Life Technologies) thrice for 5-min each time. Cells were incubated for one hour with donkey anti-rat Alexa-Fluor 488 conjugated secondary antibody (1:200 dilution; ab150153, Abcam) at room temperature. After washing with PBS, nuclei were stained with DAPI. Cells were examined using an Olympus IX81 inverted microscope with cell^F software, version 2.8 (Olympus). For the negative controls, primary antibodies were omitted (Additional file 3: Figure S2 E-H).

Immunohistochemistry and histological analysis

Three months after transplantation, recipient males were killed by cervical dislocation, the testes collected and decapsulated in DMEM/F12 and fixed in acidified formol alcohol fixative as mentioned earlier. Slides were deparaffinized in xylene and rehydrated in a descending series of isopropanol (100%, 100%, 90% and 70%) followed by a 5-min wash in phosphate buffered saline (PBS; 70011051; Life Technologies). Endogenous peroxidases were blocked in 0.3% hydrogen peroxide (H3410-500 ml; Sigma-Aldrich) for 30 min. Slides were incubated with 3% normal goat serum for 30 min which was then followed by overnight incubation with the primary mouse anti-GFP antibody (1/200; sc-9996; tebu-bio) at 4°C. The next morning, the sections were washed three times with PBS for 5 min followed by incubation with a goat anti-rabbit/mouse secondary antibody (K5007; Dako, Heverlee, Belgium) for one hour at room temperature. After three washes with PBS, 3,3’-diaminobenzidine (1/50; K5007; Dako) was added to visualize the immunoreactivity. All slides were counterstained with haematoxylin. The sections were dehydrated in a mounting series of alcohol (70%, 90%, 100% and 100%) and xylene. Finally, slides were mounted using acrytol mounting medium (100406; Surgipath, Labonord) and analyzed under an Olympus IX 81 inverted bright field microscope. Adult GFP^+^ and adult GFP^-^ mouse testicular tissue sections with the addition of primary mouse anti-GFP antibody were used as positive and negative controls, respectively. Thirty serial cross-sections per testis (with a 100µm shift between each slide) were blindly analyzed to assess the overall TFI (percent of tubules containing spermatogenesis) and the donor-derived TFI (percent of tubules containing donor-derived spermatogenesis) [27].

Double immunofluorescent staining was performed for RFP (MSCs) and MVH (Germ cell), SOX9 (Sertoli cell) or STAR (Leydig cell) to evaluate the expression of testicular cell markers by transplanted MSCs. For this purpose, rehydrated sections were incubated for 30 min in 3% (v/v) H_2_O_2_ in methanol to inhibit endogenous peroxidase activity, followed by antigen retrieval in a waterbath at 95°C in 10 mM citrate buffer (pH 6.0) containing 0.05% (v/v) Tween-20 at for 10 min. An incubation of 30 min was allowed to block non-specific binding sites in Tris-buffered saline (TBS) containing 20% (v/v) normal chicken serum (NCS; Sigma-Aldrich) and 5% (w/v) bovine serum albumin (BSA; Sigma-Aldrich), hereafter referred to as TBS/NCS/BSA. For double staining with antibodies from the same species, the sections were incubated twice overnight at 4°C with two different primary antibodies diluted in TBS/NCS/BSA. Then, slides were incubated with the first primary antibody overnight. On the second day, the slides were incubated with the secondary antibody (chicken anti-rabbit diluted 1/200 in TBS/NCS/BSA) for 30 min, followed by incubation with tyramide for 4 min (TSA Plus Fluorescein System, PerkimElmer Life & Analytical Sciences, Zaventem, Belgium) according to the manufacturer’s guidelines. Then the second round of antigen retrieval was carried out as mentioned earlier followed by washing twice with TBS and blocking with 3% H_2_O_2_ and 0.1% Tween 20 for 30 min. After washing with TBS again blocking with TBS/NCS/BSA for 30 was followed. Then, the second primary antibody was added and slides were left for incubation overnight. On day three, the slides were again incubated with secondary antibody (chicken anti-rabbit diluted 1/200 in TBS/NCS/BSA) for 30 min, followed by tyramide for 4 min (TSA Plus Cy3 System). After each step, slides were washed thrice in PBS. Finally, slides were mounted with slowfade antifade with DAPI and a coverslip was sealed with nail polish. Details of the primary and secondary antibodies and their targets are provided (Additional file 6: Table S2).
