# Supplementary material for: Co-transplantation of mesenchymal stem cells improves spermatogonial stem cell transplantation efficiency in mice
Source: Stem Cell Res Ther. 2018 Nov 21;9:317. doi: 10.1186/s13287-018-1065-0 (PMC6249754; doi:10.1186/s13287-018-1065-0)
Supplement: Supplementary file 5 — Table S1. Cytokines studied by the antibody array. (DOCX 37 kb) [file 13287_2018_1065_MOESM5_ESM.docx]

Additional file 5: Table S2. Cytokines studied by the antibody array.

| Name | Full name | UniProt mouse protein accession number |
| --- | --- | --- |
| Axl | Tyrosine-protein kinase receptor UFO | Q00993 |
| Bfgf | Fibroblast growth factor 2 | P15655 |
| BLC (CXCL13) | B lymphocyte chemoattractant | O55038 |
| CD30 Ligand (TNFSF8) | Tumor necrosis factor (Ligand) superfamily, member 8 | Q544U1 |
| CD30 (TNFRSF8) | Tumor necrosis factor ligand superfamily member 8 | P32972 |
| CD40 (TNFRSF5) | Tumor necrosis factor receptor superfamily member 5 | P27512 |
| CRG 2 | C-X-C motif chemokine 10 | P17515 |
| CTACK (CCL27) | C-C motif chemokine 27 | Q9Z1X0 |
| CXCL16 | C-X-C motif chemokine 16 | Q8BSU2 |
| CD26 (DPPIV) | Dipeptidyl peptidase 4 | P28843 |
| Dtk | Tyrosine-protein kinase receptor TYRO3 | P55144 |
| Eotaxin 1 (CCL11) | C-C motif chemokine 11 | P48298 |
| Eotaxin 2 (CCL24) | C-C motif chemokine 24 | Q68Y86 |
| E-Selectin | E-selectin | Q00690 |
| Fas Ligand (TNFSF6) | Tumor necrosis factor ligand superfamily member 6 | P41047 |
| Fc gamma RIIB (CD32b) | Low affinity immunoglobulin gamma Fc region receptor II | P08101 |
| Flt 3 Ligand | Fms-related tyrosine kinase 3 ligand | P49772 |
| Fractalkine (CX3CL1) | Fractalkine | O35188 |
| GCSF | Granulocyte colony-stimulating factor | P09920 |
| GITR (TNFRSF18) | Tumor necrosis factor receptor superfamily member 18 | O35714 |
| GM-CSF | Granulocyte-macrophage colony-stimulating factor | P01587 |
| HGFR | Hepatocyte growth factor receptor | P16056 |
| ICAM-1 (CD54) | Intercellular adhesion molecule 1 | P13597 |
| IFN-gamma | Interferon gamma | P01580 |
| IGFBP 2 | Insulin-like growth factor-binding protein 2 | P47877 |
| IGFBP 3 | Insulin-like growth factor-binding protein 3 | P47878 |
| IGFBP 5 | Insulin-like growth factor-binding protein 5 | Q07079 |
| IGFBP 6 | Insulin-like growth factor-binding protein 6 | P47880 |
| IGF 1 | Insulin-like growth factor I | P05017 |
| IGF 2 | Insulin-like growth factor II | P09535 |
| IL 1 beta (IL 1 F2) | Interleukin-1 beta | P10749 |
| IL 10 | Interleukin-10 | P18893 |
| IL 12 p40/p70 | Interleukin-12 beta | P43432 |
| IL 12 p70 | Interleukin-12 alpha | P43431 |
| IL 13 | Interleukin-13 | P20109 |
| IL 15 | Interleukin-15 | P48346 |
| IL 17A | Interleukin-17A | Q62386 |
| IL 17 RB | Interleukin-17 receptor B | Q9JIP3 |
| IL 1 alpha (IL 1 F1) | Interleukin-1 alpha | P01582 |
| IL 2 | Interleukin-2 | P04351 |
| IL 3 | Interleukin-3 | P01586 |
| IL 3 R beta | Cytokine receptor common subunit beta | P26955 |
| IL 4 | Interleukin-4 | P07750 |
| IL 5 | Interleukin-5 | P04401 |
| IL 6 | Interleukin-6 | P08505 |
| IL 7 | Interleukin-7 | P10168 |
| IL 9 | Interleukin-9 | P15247 |
| I TAC (CXCL11) | C-X-C motif chemokine 11 | Q9JHH5 |
| KC (CXCL1) | Growth-regulated alpha protein | P12850 |
| Leptin | Leptin | P41160 |
| Leptin R | Leptin receptor | P48356 |
| LIX | Protein limb expression 1 homolog | Q6P566 |
| L Selectin (CD62L) | L-selectin | P18337 |
| Lungkine (CXCL15) | C-X-C motif chemokine 15 | Q9WVL7 |
| Lymphotactin (XCL1) | Lymphotactin | P47993 |
| MCP 1 (CCL2) | C-C motif chemokine 2 | P10148 |
| MCP 5 (CCL12) | C-C motif chemokine 12 | Q62401 |
| M CSF | Macrophage colony-stimulating factor | P07141 |
| MDC (CCL22) | C-C motif chemokine 22 | O88430 |
| MIG (CXCL9) | C-X-C motif chemokine 9 | P18340 |
| MIP 1 alpha (CCL3) | C-C motif chemokine 3 | P10855 |
| MIP 1 gamma | C-C motif chemokine 9 | P51670 |
| MIP 2 | C-X-C motif chemokine 2 | P10889 |
| MIP 3 beta (CCL19) | C-C motif chemokine 19 | O70460 |
| MIP 3 alpha (CCL20) | C-C motif chemokine 20 | O89093 |
| MMP 2 | 72 kDa type IV collagenase | P33434 |
| MMP 3 | Stromelysin-1 | P28862 |
| Osteopontin (SPP1) | Osteopontin | P10923 |
| Osteoprotegerin (TNFRSF11B) | Tumor necrosis factor receptor superfamily member 11B | O08712 |
| Platelet Factor 4 (CXCL4) | Platelet factor 4 | Q9Z126 |
| Pro-MMP 9 | Pro-MMP-9 | Q91ZS5 |
| P-Selectin | P-selectin | Q01102 |
| RANTES (CCL5) | C-C motif chemokine 5 | P30882 |
| Resistin | Resistin | Q99P87 |
| SCF | Kit ligand | P20826 |
| SDF 1 alpha | Stromal cell-derived factor 1 | P40224 |
| Shh N | Sonic hedgehog N-Terminal protein | Q62226 |
| TNF RI (TNFRSF1A) | Tumor necrosis factor receptor superfamily member 1A | P25118 |
| TNF RII (TNFRSF1B) | Tumor necrosis factor receptor superfamily member 1B | P25119 |
| TARC (CCL17) | C-C motif chemokine | F6R5P4 |
| I-309 (TCA 3/CCL1) | C-C motif chemokine 1 | P10146 |
| TECK (CCL25) | C-C motif chemokine 25 | O35903 |
| TCK 1 (CXCL7) | Chemokine (C-X-C motif) ligand 7 | Q9EQI5 |
| TIMP 1 | Metalloproteinase inhibitor 1 | P12032 |
| TIMP 2 | Metalloproteinase inhibitor 2 | P25785 |
| TNF alpha | Tumor necrosis factor alpha | P06804 |
| Thrombopoietin (TPO) | Thrombopoietin | P40226 |
| TRANCE (TNFSF11) | Tumor necrosis factor ligand superfamily member 11 | O35235 |
| TROY (TNFRSF19) | Tumor necrosis factor receptor superfamily member 19 | Q9JLL3 |
| TSLP | Thymic stromal lymphopoietin | Q9JIE6 |
| VCAM 1 (CD106) | Vascular cell adhesion protein 1 | P29533 |
| VEGF A | Vascular endothelial growth factor A | Q00731 |
| VEGFR1 | Vascular endothelial growth factor receptor 1 | P35969 |
| VEGFR2 | Vascular endothelial growth factor receptor 2 | P35918 |
| VEGFR3 | Vascular endothelial growth factor receptor 3 | P35917 |
| VEGF D | Vascular endothelial growth factor D | P9794 |
