# Supplementary material for: Co-transplantation of mesenchymal stem cells improves spermatogonial stem cell transplantation efficiency in mice
Source: Stem Cell Res Ther. 2018 Nov 21;9:317. doi: 10.1186/s13287-018-1065-0 (PMC6249754; doi:10.1186/s13287-018-1065-0)
Supplement: Supplementary file 6 — Table S2. Antibodies used for immunocytochemistry and immunohistochemistry. (DOCX 32 kb) [file 13287_2018_1065_MOESM6_ESM.docx]

**Additional file 1: Table S1. Antibodies**

| **Target protein** | **Full Name** | **Host** | **Dilution** | **Source** |
| --- | --- | --- | --- | --- |
| MVH | Mouse vasa homologue | Rabbit polyclonal | 1:200 | Abcam (Ab13840) |
| SOX9 | Sex determining region Y-box 9 | Rabbit polyclonal | 1:200 | Millipore, Overijse, Belgium (Ab5535) |
| STAR | Steroidogenic acute regulatory  protein | Rabbit polyclonal | 1:500 | Santa-Cruz biotechnology, (SC-25806) |
| UCHL1 | Ubiquitin carboxyl-terminal  esterase L1 | Rabbit polyclonal | 1:500 | Bio-Rad (Formerly AbD Serotec)  (7863-0504) |
| GFP (IHC) | Green fluorescent protein | Mouse monoclonal | 1:100 | Santa Cruz (sc-9996) |
| RFP | Red fluorescent protein | Rabbit polyclonal | 1:100 | Abcam (Ab62341) |
| CD44 | Cluster differentiation antigen 44 (homing cell adhesion molecule) | Rat monoclonal | 1:500 | Abcam (Ab25340) |
| CD45 | Cluster of differentiation 45 (Protein tyrosine phosphatase, receptor type, C) | Rat monoclonal | 1:500 | Abcam (Ab25386) |
| Sca1 | Stem cells antigen 1 | Rat monoclonal | 1:500 | Abcam (Ab51317) |
| CD29 | Cluster of differentiation 29 (Integrin beta 1) | Rat monoclonal | 1:500 | Abcam (Ab95623) |
| Alexa-Fluor 488 | NA | Anti-rabbit | 1:200 | A21206; Life Technologies, Merelbeke, Belgium |
| Alexa-Fluor 488 | NA | Anti-rat | 1:200 | Abcam, ab150153; Cambridge, UK |
| Chicken anti-rabbit IgG | NA | Anti-rabbit | 1:200 | Santacruz Biotechnology (SC-2963) |
